# Use of Hyd. Potass. in Hydrocephalus

**Published:** 1847-06

**Authors:** Thompson Mead

**Affiliations:** Batavia, Kane county, Illinois


ILLINOIS AND INDIANA

MEDICAL AND SURGICAL JOURNAL.

Vol. II NEW SERIES.—JUNE, 1847. No. 2.

### PART I.—ORIGINAL COMMUNICATIONS.

### ARTICLE I.

*Use of Hyd. Potass. in Hydrocephalus.* By THOMPSON MEAD, M. D., of Batavia, Kane county, Illinois.

The following cases of Hydrocephalus—the symptoms of which were well marked and decided in their character—were wholly treated by Hyd. Potass., unless there were some complications which required other means. Without detailing the symptoms as they occurred from day to day, I shall only mention the dose, &c., and the result. By saying they were wholly treated by Hyd. Potass., I mean that my entire dependence was in this, though, to some extent, other means were made use of.

CASE I—*June* 16*th*, 1842.—W. B., aged 5 years, strumous habit. The case was considered hopeless. The bowels having been moved by a calomel purge, I resorted to Hyd. Potass., and gave it in substance in doses of three grains every two and a half hours—the application of cold and blisters still continued. After the third dose, the pulse became slower and fuller and heat more equal.

*June* 17*th*.—The patient could be roused. Heat and circulation much more equalized; scarcely any appearance of convulsions or strabismus; free secretion of urine, which was dark and rather fetid; eye sensible to light and pupil more natural. I gradually reduced the size of the dose and increased the intervals. On the fourth day I considered the patient out of danger. There was no other medicine given except an occasional dose of oil to move the bowels.

Case II—*July* 10*th*, 1843.—A. H., aged 6 years, stroumous habit. This case was considered incurable, and the attending physician had discontinued his visits. The bowels had been freely moved by salts and senna. I gave three grains of Hyd. Potass. every three hours; continued the counter irritation, cold to the head, as the head was hot, &c.; and gave calomel and ipecac. once in three hours, in the intervals of giving the Potass.

*July* 12*th*.—Perfect restoration of consciousness, though complains of some pain in the head; free discharge of urine; bowels more open; extremities warm. In short, the disease entirely subsided under this course. On the 12th, the mouth became sore with dribbling of saliva. There was none of that fetor characteristic of a calomel sore mouth. On the 15th I considered him out of danger.

Case III—*December* 20*th*, 1845.—J. R., aged 4 years, strumous habit. This case was complicated with some mesenteric disease, and was considered incurable—the attending physician having discontinued his visits. The original attack was pneumonia, and attended with considerable derangement of the bowels. As the pneumonia subsided, the trouble in the brain supervened. I gave the Potass. in substance, in doses of three grains every three hours, and a pill of calomel, ipecac. and quinine once in three hours in the intervals of giving the potass. On the 25th the coma, &c., had entirely subsided, as well as all disposition to convulsions; a free discharge of urine; throat and mouth sore. There was considerable tenderness of the abdomen; evacuations from the bowels dark and fetid; general restlessness. I added morphine to the pill and gave it once in six hours, and also made use of counter irritation. He remained weak and irritable for some time but gradually recovered. Appetite became natural, bowels regular, and the enlargement of the abdomen finally entirely subsided. I continued through the entire course the potass. in moderate doses—towards the last I gave it in solution.

Case IV—*November 20th*, 1846.—J. W., aged 19 years, strumous habit. This patient had been sick for some time before I saw her, and I inferred, from the account I received of her previous sickness, that the effusion followed congestion of the brain as a consequence of chills, &c. It was complicated with considerable hepatic disease. For two or three periods the catamenia had been almost entirely suppressed, and the chills had been followed by more or less stupor; and on one occasion the entire body was covered with purple spots. At this time the stupor was not immediately preceded by a chill. She had complained for some weeks previous of pain, dizziness, a feeling of emptiness in her head, a sensation of falling forwards which was more or less constant, and now and then double vision. About November 12th, there was evidence of hepatic disease—the liver could be felt considerably enlarged at the time I first saw her. On the 25th a severe hæmorrhage occurred from the bowels, which, however, was easily controlled by Tannin. This was followed by a discharge of pus of a greenish yellow appearance. These discharges prostrated her considerably, and immediately following this last evacuation the tenderness, &c., over the liver subsided.

*November 20th.*—The urine has been suppressed for some three days. I applied a blister on the neck and on the temples, kept the one over the liver open, and applied mustard to the extremities. I gave her what I judged to be ten grains Hyd. Potass. every three hours, and a pill of—

℞.—Ext. Tarax. gr. iij.;

Sulph. Quinia gr. j.;

Calomel gr. $\frac{1}{2}$;

Morphine gr. $\frac{1}{4}$; every six hours.

*November 23d.*—My case book reads: "Better than has been; a free discharge of urine which was dark, &c.; pulse 98 (had been up to 118); extremities warmer; eyes somewhat sensible to light; pupils not so much dilated; countenance less expressive of suffering; relaxation of the flexors, &c."

The bowels not having moved off as much as was desirable, I ordered crot. oil and soap in the form of a pill once in two hours till it should operate.

*November 25th.*—The pulse was reduced to 88; urine not so dark as on the 23d; the oil had freely moved the bowels; as the stupor subsided she evinced symptoms of mental derangement; pupils rather contracted. After the hæmorrhage from the bowels, I omitted the calomel, and increased the amount of quinine.

*November 27th.*—Better; tongue cleaning; bowels regular. From this time forward she steadily improved, the mental derangement gradually subsided, and all the functions were healthily performed.

In all cases after the dribbling of saliva commenced, I diminished the dose materially, and in some cases had to omit it for a time. These cases have been taken at random from among others in which I have tested the value of this agent, and in all the cases I have mentioned all the ordinary means had been made use of, and without any appreciable benefit, with the exception of the case of L. W. I am confident it is a remedial means of no ordinary value in this disease. The most marked effect in all the cases in which I have given it was diuresis, dribbling of saliva, and generally the rapid disappearance of the convulsions, and the insensibility.

The conditions of the brain in which I have mostly used this agent are *Hydrocephalus* and *Hypercæmia*. For the former I consider it as much of a specific as anything in medicine can be. That it will generally cure unless dependent on organic disease I aver—that it will occasionally fail I admit. *Hydrocephalus*, in a majority of cases, attacks patients in whom the strumous diathesis is predominant, and, if any reliance is to be placed in the pathological investigations of *Dana*, *Gerhard*, *Greene*, *Guersent*, *Becquerel*, and others, that the effusion is caused by a "deposition of tubercular granulations on the surface of the cerebral layer of the arachnoid membrane," in all cases, why may not its modus operandi consist in effecting some change in those granulations, even if they do not cause their absorption, and in checking that diseased action on which those deposits depend? We know full well that Hyd. Potass. has singular control over strumous affections. When it is liberally given, as it should be in such cases, heat and circulation become equalized, all the functions become healthy, but the kidneys are the first to resume their wonted activity.

About the time the heat and circulation become equalized the throat and mouth become sore—a species of ptyalism comes on, yet different from that which is occasioned by calomel. I have noticed this to a greater or less extent in all the cases which I have seen recover by the use of this agent; and I have no recollection of a case which recovered when it did not appear. I have, therefore, been led to consider it as an evidence of its *specific* or *constitutional effect*. I have seen it restore the heat in the extremities, and, to some extent, increase the amount of urine, and exert a controlling influence over the convulsions, &c., and yet the patients sink under the disease—the mouth and throat showing no evidence of soreness. If it cannot be considered an evidence of its specific or constitutional effect, it certainly must be regarded as a curious coincidence. I have not observed any injurious effect from it on the stomach and bowels, even when given in large doses and frequently repeated.

*Hydrocephalus* has been one of the *opprobria medicinæ*, and I am convinced that it will not much longer be so if physicians would give Hyd. Potass. a thorough trial; though not to the exclusion of some of the ordinary means, such as counter irritation, cold to the head when necessary, keeping the bowels freely open, &c.

In getting at the true value of a medicinal agent, it is the duty of a physician to take into consideration its effects on the animal economy in health, the various conditions of the system in disease, and the circumstances by which the patient may be surrounded, which will, to a greater or less extent, modify its effect on the organization. The opinions that may be entertained of the remedial effect of any particular article of the Materia Medica unless its operation is investigated in this manner, are very often erroneous, and this diversity of opinion has only arisen from the too careless manner of tracing the relation of cause and effect, to the extent it can be in medicine. Medical evidence must of necessity be cumulative — when one fact is indisputably established, it is material either in strengthening or refuting theories, and becomes the foundation for other improvements.

*January 15th,* 1847.

[We have taken the liberty of publishing the above with the author's name, for which we hope he will excuse us. We could not suppress it, and we have adopted the rule of publishing no anonymous article.—*Editors.*]

